# Donkey milk-derived exosomes protect against UVB irradiation-induced ferroptosis in skin cells: *in vitro* and *in vivo* evidence

**DOI:** 10.3389/fphar.2025.1683253

**Published:** 2026-01-02

**Authors:** Jie Yu, Jie Cheng, Guangyuan Liu, Zhijie Cheng, Pengxiang Niu, Derui Xu, Xinyun Pei, Hang Tie, Cong Wang

**Affiliations:** 1 Dong-E E-Jiao Co. Ltd., Liaocheng, Shandong, China; 2 Tianjin Key Laboratory of Animal and Plant Resistance, College of Life Sciences, Tianjin Normal University, Tianjin, China; 3 Chinese Academy of Inspection & Quarantine Greater Bay Area, Zhongshan, Guangdong, China

**Keywords:** donkey milk-derived exosomes, ferroptosis, ROS, skin damage, UVB

## Abstract

UVB irradiation can induce ferroptosis and accelerates skin photoaging. However, the role of donkey milk-derived exosomes (DM-Exos) on UVB induced ferroptosis was unclear. In this study, using HaCaT keratinocytes and CCC-ESF-1 fibroblasts exposed to UVB irradiation (60–100 mJ/cm^2^), we found that UVB irradiation significantly reduced skin cell viability, while DM-Exos treatment effectively reversed this decline. To investigate the underlying mechanism, we assessed key markers of ferroptosis, including ROS, lipid peroxides (LipoROS), malondialdehyde (MDA), glutathione (GSH), 4-hydroxynonenal (4-HNE), glutathione peroxidase 4 (GPX4), and ferritin heavy chain 1 (FTH1) and the results showed that UVB irradiation increased the levels of ferroptosis-related biomarkers. DM-Exos treatment reversed these changes, suggesting its role in mitigating ferroptosis. Furthermore, in a UVB-induced photoaging mouse model, subcutaneous administration of DM-Exos ameliorated skin damage, improved hydration, and reduced ferroptosis biomarkers in dorsal skin. These findings establish DM-Exos as a novel biological agent against UVB-induced skin injury and delineate a previously unrecognized mechanism linking milk-derived exosomes to ferroptosis regulation.

## Highlights

- Protective Effects of Donkey Milk Exosomes Against UVB Irradiation-Induced Skin Cell Damage.
- Donkey Milk Exosomes Suppress Ferroptotic Pathways in Skin Cells.
- Application of Donkey Milk Exosomes Ameliorates UVB-Induced Damage *in vivo*.

## 1 Introduction

Ultraviolet B (UVB) radiation is a key component of solar ultraviolet (UV) light, with a wavelength range of 280–315 nm, falling between UVA (315–400 nm) and UVC (100–280 nm) (Labecka et al., 2024; Raza et al., 2024; Yang et al., 2023). Acute UVB overexposure leads to sunburn (erythema, edema) and melanin deposition (tanning), while chronic exposure accelerates photoaging, characterized by wrinkles, loss of elasticity, and an increased risk of non-melanoma skin cancers, such as basal cell carcinoma and squamous cell carcinoma (Zhang et al., 2024). At the molecular level, UVB irradiation can trigger multiple types of programmed cell death, including apoptosis, autophagic cell death, pyroptosis, ferroptosis, and necroptosis (Chen Y. et al., 2024; Li et al., 2021; Newton et al., 2024; Zhang et al., 2017). Recent studies have shown that the UVB-induced skin damage and photoaging may be achieved through triggering ferroptosis in cells (Vats et al., 2021; Feng et al., 2022). The essence of ferroptosis is depletion of cysteine, which leads to deficit of reduced GSH (Tang et al., 2021), toxic lipid alkoxy radicals in free iron-mediated Fenton-type reactions that disrupt the balance of the intracellular redox state, resulting in oxidative stress and lipid peroxidation both in the cell plasma membrane and in membranes of other internal organelles, triggering cell death (Tang et al., 2021). Thus, targeting ferroptosis inhibition could be a promising approach to mitigating UVB-induced skin damage.

Extracellular vesicles (EVs) including exosomes, microvesicles, and apoptotic vesicles, are membrane-bound structures secreted by cells; they contain various bioactive components, including proteins, lipids, nucleic acids, and both coding and non-coding RNA derived from donor cells (Kalluri, 2024). Considering the effects of UV radiation, exosomes derived from mesenchymal stem cells (MSC-Exos) were used to counteract oxidative stress and skin aging by degrading MMPs and down-regulating the production of reactive oxygen species (Tienda-Vázquez et al., 2023). A growing body of literature confirms that exosomes are ubiquitous in the milk of various mammals, including bovine milk (Cui et al., 2023; Yang et al., 2024; Zempleni et al., 2019), human breast milk (Akinduro et al., 2025; Di et al., 2024), and porcine milk (Chen et al., 2016). Notably, recent research has identified the presence of extracellular exosomes, typically sized between 30 and 150 nm, in donkey milk, which are posited to play significant roles in promoting antioxidants (Liu et al., 2025). Emerging data suggest that DM-Exos have 2293 proteins and 212 miRNAs were identified in donkey milk by 4D label-free quantitative proteomics and Illumina sequencing technology, respectively (Liu et al., 2025; Shang et al., 2023). Thus, DM-Exos have gained increasing attention for its potential role in protecting skin from UVB-induced oxidative damage.

This study investigates DM-Exos as a potential intervention for UVB-induced oxidative damage and ferroptosis in skin cells. Specifically, we used HaCaT keratinocytes and CCC-ESF-1 fibroblasts exposed to UVB irradiation (60–100 mJ/cm^2^) to establish *in vitro* models of photodamage. In parallel, a UVB-induced photoaging mouse model was established, and DM-Exos were administered via subcutaneous injection to evaluate their therapeutic efficacy *in vivo*. We first examined the effects of UVB irradiation on skin cell viability and then evaluated key ferroptosis markers, including ROS, lipoROS, GSH, MDA, 4-HNE, GPX4, FTH1 *in vitro* and *in vivo*. Our findings confirm that DM-Exos mitigate UVB-induced skin damage by reducing lipid peroxidation, preserving ferroptosis-regulating proteins, and limiting DNA damage. Collectively, the present study successfully extracted and prepared DM-Exos and suggested a promising exosome-based strategy for anti-ferroptosis therapy in UVB-induced skin damage.

## 2 Materials and methods

### 2.1 Cell culture

Human skin epidermal keratinocytes (HaCaT) and dermal fibroblasts (CCC-ESF-1) were supplied by the Cell Resource Center of Institute of Basic Medical Sciences, Chinese Academy of Medical Sciences. HaCaT cells were cultured in Minimum Essential Medium (MEM) (Gibco, Life Technologies, Carlsbad, CA, United States of America) supplemented with 15% fetal bovine serum (FBS) (ExCell, Suzhou, China) and 1% penicillin-streptomycin (Gibco). CCC-ESF-1 cells were maintained in Dulbecco’s Modified Eagle Medium (DMEM) with the same supplements. Both cell lines were maintained at 37 °C in a humidified incubator of 5% CO_2_ and 95% air.

### 2.2 Isolation and characterization of DM-Exos and MSC-Exos

Exosomes were isolated from donkey milk and mesenchymal stem cell (MSC) conditioned medium using established differential ultracentrifugation protocols with appropriate modifications (Liu et al., 2025; Kore et al., 2021). Isolation of DM-Exos: 200 mL of donkey milk was used as the starting material for the exosome isolation procedure. Donkey mature milk (30 days postpartum) sample were first centrifuged at 2,000 g for 30 min at 4 °C, followed by further centrifugation of the supernatant at 10,000 g for 45 min at 4 °C. The collected supernatant was filtered through a 0.45 μm membrane, and the resulting filtrate was ultracentrifuged at 100,000 g for 70 min at 4 °C. After discarding the supernatant, the pellet was resuspended in 1 mL of ice-cold 1× PBS buffer. This suspension was layered onto a 250 μL 30% sucrose cushion and ultracentrifuged again at 100,000 × g for 70 min. The interphase fraction (above the sucrose cushion) was collected and subjected to another ultracentrifugation at 100,000 g for 70 min. The final exosome pellet was resuspended in 500 μL PBS for storage at −80 °C. Isolation of MSC-Exos: Human umbilical cord-derived MSCs were cultured in serum-free DMEM for 16–18 h. The conditioned medium was collected and sequentially centrifuged at 3,000 g for 10 min and 10,000 g for 30 min to remove cells and debris. The supernatant was then ultracentrifuged at 100,000 g for 3 h, and finally resuspended in PBS. The total protein concentration of the purified exosome pellet, was determined using the BCA assay. Exosomal morphology and particle size was characterized using the transmission electron microscopy (TEM), and nanoparticle tracking analysis (NTA), respectively.

### 2.3 Cellular uptake

HaCaT and CCC-ESF-1 cells were seeded in 24-well plates and cultured for 24 h under standard conditions. Subsequently, the cells were treated with DiO-loaded Exos and incubated for an additional 12 h. DiO-labeled positive cells were visualized by fluorescence microscope.

### 2.4 Establishment of UVB-Induced skin photoaging mouse model and exosomes treatment

Four to six-week-old female Kunming mice were purchased from Chengdu Dashuo Experimental Animal Company (Chengdu, China). Skin wrinkles were induced using a UVB lamp following previously established methods (Chen J. et al., 2024; Sun et al., 2024). The minimal erythemal dose (MED) was set at 300 mJ/cm^2^, with irradiation applied in increasing doses each week: Week 1: 1 MED (300 mJ/cm^2^), Week 2: 2 MED (600 mJ/cm^2^), Week 3: 3 MED (900 mJ/cm^2^), Weeks 4–8: 4 MED (1,200 mJ/cm^2^). Following UVB irradiation, 20 μg of DM-Exos and MSC-Exos were administered via subcutaneous injection into the dorsal skin of mice three times per week. Normal control mice received subcutaneous injections of the same volume of saline on the same schedule. At the end of the 8-week period, mice were euthanized, and dorsal skin samples and blood (collected via orbital venipuncture) were harvested for further analysis.

### 2.5 UVB exposure of skin cells

HaCaT and CCC-ESF-1 cells were washed twice with PBS and irradiated with UVB in serum-free medium. UVB exposure was delivered using a bank of TL 20 W/20 fluorescent tubes (Philips, wavelength range: 290–320 nm, peak 312 nm) at an intensity of 2.2 mW/cm^2^ for 9 min. The total UVB radiation dose was 20 mJ/cm^2^, corresponding to an average exposure of 1.52 × 10^−3^ mJ per cell. During irradiation, the dish lids were removed, and the UVB dose was adjusted by modifying the lamp-to-cell distance and irradiation time to ensure precise exposure control.

### 2.6 Cell viability assay

Cell viability following UVB irradiation and compound treatment was assessed using the Cell Counting Kit-8 (CCK-8) assay, following the manufacturer’s instructions. HaCaT and CCC-ESF-1 cells were seeded in 96-well plates at a density of 5,000 cells per well. For experiments involving cell death pathway inhibitors, cells were treated with Z-VAD-FMK (apoptosis inhibitor, 20 µM), Nec-1 (necroptosis inhibitor, 50 µM), or Fer-1 (ferroptosis inhibitor, 2 µM). After the treatment, the culture medium was removed, and CCK-8 reagent was added to each well. After incubation for 2 h, the absorbance of the plate was measured at 450 nm.

### 2.7 Measurement of intracellular ROS and lipid ROS

Cells were seeded in 24-well plates and treated according to the indicated experimental conditions. After treatment, the medium was removed, and cells were incubated with 10 μM DCFH-DA or 5 μM BODIPY 581/591 C11 in serum-free medium. ROS and Lipid ROS levels were visualized via confocal microscopy.

### 2.8 Mitochondrial membrane potential assay

Mitochondrial membrane potential was measured using Rhodamine 123 (Rho123) (Beyotime, China). The probe was diluted in serum-free medium (1:2500, final concentration 2 µM). Cells were washed with serum-free medium, incubated with diluted Rho123 for 30 min, and then washed three times to remove unbound probe. Fluorescence images were captured using a confocal microscope.

### 2.9 Transmission electron microscopy of mitochondrial morphology

Cells were fixed with 2.5% glutaraldehyde in phosphate buffer and subsequently treated with 1% OsO_4_ for 2 h. After dehydration, the cells were embedded in epoxy resin. Ultrathin sections were prepared using an ultramicrotome, stained with lead citrate and uranyl acetate, and the distribution of mitochondria was observed using a transmission electron microscope.

### 2.10 MDA measurement

The MDA amounts in the supernatants from lysed cells were assessed using an MDA Assay Kit (Beyotime Institute of Biotechnology, Nantong, China) according to the manufacturer’s instructions. The fluorescence intensity was measured at 532 nm by a fluorescence reader.

### 2.11 Measurement of intracellular GSH level

HaCaT and CCC-ESF-1 cells were seeded in 6 cm dishes and incubated overnight. After UVB irradiation, cells were treated with DM-Exos -containing medium at different concentrations for 24 h. GSH and GSSG levels were measured using the GSH and GSSG Assay Kit (Beyotime, China) following the manufacturer’s instructions.

### 2.12 GPX4 and ACSL4 activity assay

For GPX4 activity detection, the cells were measured by glutathione peroxidase 4 activity assay kit (Elabscience, China), according to the manufacturer’s instructions. The ACSL4 activity was measured following a previously described methodology (Huang et al., 2024). The result was expressed as a percentage of the total enzymatic activity.

### 2.13 Quantitative real-time PCR

Total RNA was extracted with Trizol lysis buffer, and was then transformed into cDNA. Primer sequences are provided in Supplementary Table S1.

### 2.14 Immunohistochemical staining

Mice skin was harvested and fixed in 4% paraformaldehyde (PFA) in 10 mM PBS overnight at 4 °C. After paraffin embedding, 5 μm-thick skin sections were cut using a paraffin microtome. The sections were then deparaffinized using routine procedures, followed by antigen retrieval using an alkaline retrieval solution in a pressure cooker for 2 min. To block endogenous peroxidase activity, tissue sections were washed with PBS for 5 min, then incubated with 0.3% H_2_O_2_ in PBS for 10 min at 25 °C. After another 5-min PBS wash, sections were incubated with 10% normal goat serum for 30 min at room temperature to block nonspecific binding. Sections were then incubated overnight at 4 °C with the primary antibody, followed by HRP-labeled goat anti-rabbit IgG at a 1:500 dilution for 2 h in the dark. After washing, DAB substrate was applied for 2 min at room temperature to visualize staining. Tissue sections were then counterstained with hematoxylin for 40 s and dehydrated through a graded alcohol series.

### 2.15 Hematoxylin and eosin (H&E) staining

Experimental mice were euthanized by decapitation, and the dorsal skin was carefully harvested and cut into appropriate sizes. Skin samples were fixed in 10% formalin for 48 h, followed by dehydration and paraffin embedding. Paraffin-embedded samples were sectioned at 4 μm, mounted on slides, and stained with hematoxylin and eosin (H&E). Sections were deparaffinized in xylene, after a 30 s wash with distilled water. Finally, slides were sealed with neutral resin, examined under a microscope, and photographed.

### 2.16 Measurement of skin hydration

6 mm-diameter skin samples were extracted from the dorsal back. Hydration of the dorsal skin surface was measured with the Mericom Hydration Meter model 1504.

Skin hydration was assessed using the Mericom Hydration Meter (Model 1504). Dorsal skin samples (6 mm in diameter) were collected and measured according to the manufacturer’s instructions.

### 2.17 Skin lesion scoring method

Skin lesions in mice were evaluated based on clinical scoring criteria for dermatological diseases: Grade 0 (Normal): Normal skin structure without pathological changes; Grade 1 (Mild injury): Mild pathological changes with slight erythema, occasional and minor scratching, minimal scabbing, no edema, and mild inflammatory response; Grade 2 (Moderate injury): Moderate pathological changes with larger areas of erythema, intermittent scratching, partial scabbing, little to no edema, and a noticeable inflammatory response; Grade 3 (Severe injury): Severe pathological changes with extensive and dense erythema, frequent scratching accompanied by edema, and a strong inflammatory response. Scoring was conducted by assessing the skin condition of each mouse according to these criteria.

### 2.18 Statistical analysis

All data are presented as the mean ± standard deviation (SD) and are representative of at least three independent experiments. Statistical significance between control and treatment groups was determined using GraphPad Prism 8 software (GraphPad Software, La Jolla, CA, United States of America). Comparisons between two groups were performed using the unpaired two-tailed Student’s t-test, while multiple group comparisons were conducted using one-way analysis of variance (ANOVA) followed by Tukey’s *post hoc* test. A P-value <0.05 was considered statistically significant.

## 3 Results

### 3.1 Donkey milk-derived exosomes ameliorate UVB-induced damage in skin cells

**FIGURE 1:**
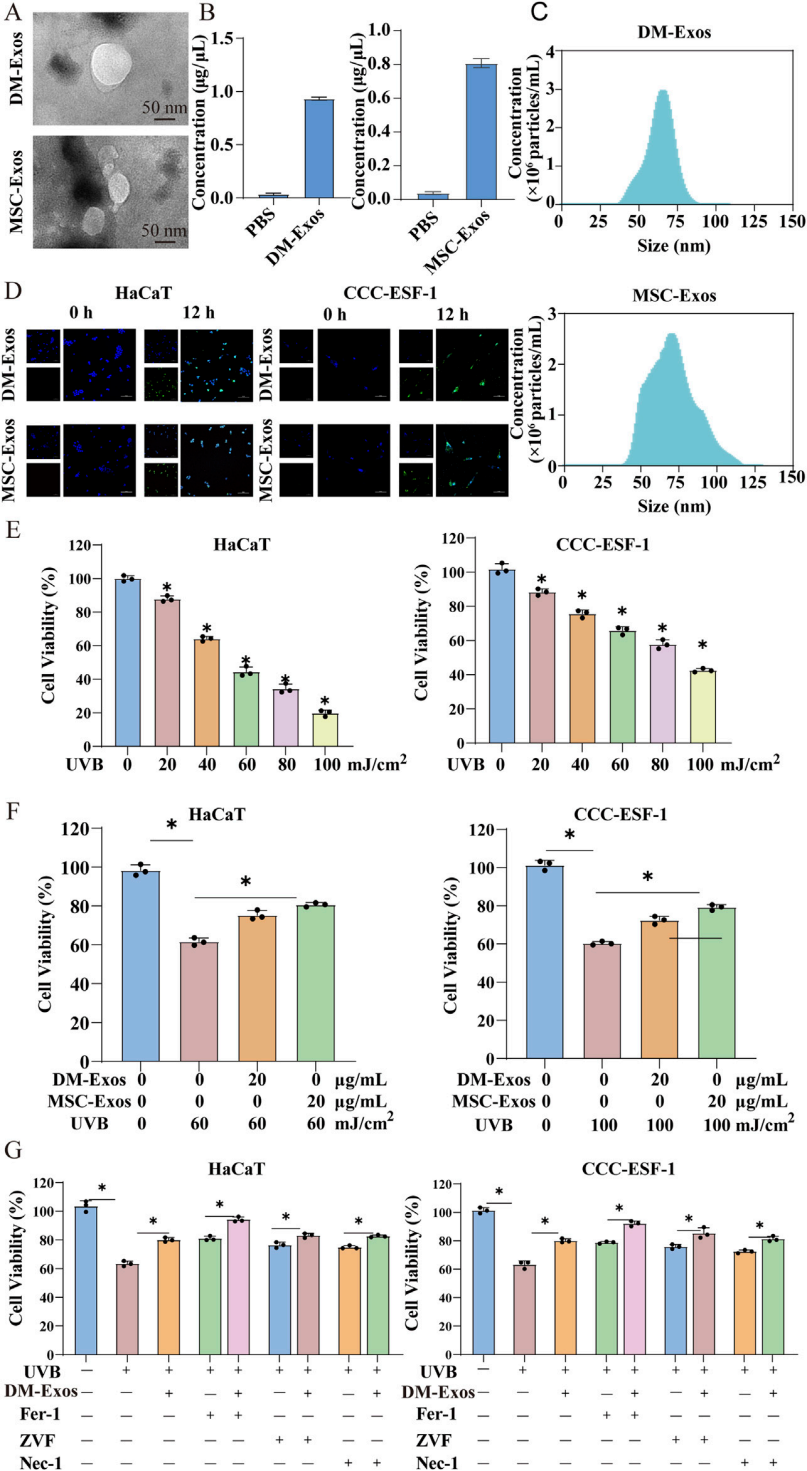
Donkey milk-derived exosomes ameliorate UVB-induced damage in skin cells. **(A)** TEM observation of DM-Exos and MSC-Exos. **(B)** Box plots of protein concentration in DM-Exos and MSC-Exos. **(C)** Particle diameter distribution was analyzed by NanoFCM. **(D)** Representative fluorescence images of Dio-labeled DM-Exos and MSC-Exos in HaCaT and CCC-ESF-1 cells. **(E)** Dose-dependent effect of UVB irradiation on cell viability. **(F)** Effect of DM-Exos on UVB-irradiated cells. **(G)** Effects of ferroptosis, apoptosis, necroptosis inhibitors (Fer-1, Z-VAD-fmk (ZVF), or Nec-1) and DM-Exos (20 ug/mL) on UVB-irradiated cells. *Data are presented as mean ± SD, with statistical significance indicated by P < 0.05.

Initial characterization confirmed the successful isolation of biologically active exosomes from donkey milk, with transmission electron microscopy revealing typical cup-shaped vesicles (40–100 nm) possessing intact lipid bilayers and a protein concentration of 1 μg/μL (Figures 1A–C). To functionally validate the cellular uptake of exosomes, we found that both HaCaT and CCC-ESF-1 cells efficiently internalized DiO-labeled donkey milk-derived exosomes (Figure 1D). Following the successful characterization of DM-Exos, we proceeded to establish UVB-induced damage models to evaluate their protective effects. A comprehensive dose-response analysis was first performed by exposing HaCaT and CCC-ESF-1 cells (Ge et al., 2024; Zhang et al., 2018) to graded UVB irradiation (0–100 mJ/cm^2^), which revealed a dose-dependent viability reduction in both cell types (Figure 1E). Based on these findings, we selected 60 mJ/cm^2^(HaCaT) and 100 mJ/cm^2^ (CCC-ESF-1) as optimal doses for subsequent experiments, representing approximately 50% viability to ensure adequate dynamic range for rescue studies. To validate the therapeutic effects of DM-Exos on UVB-induced skin damage, MSC-Exos were employed as a positive control based on their UVB-radioprotective properties (Angelina et al., 2025) (Figures 1A–D). Treatment with DM-Exos (20 μg/mL) more effectively reversed UVB-induced cytotoxicity, compared with umbilical cord mesmesenchymal stem cells-derived exosomes (MSC-Exos, 20 μg/mL) (Figure 1F). As reports, UVB irradiation can trigger multiple types of programmed cell death, including apoptosis (Ye et al., 2025), ferroptosis (Deng et al., 2023), and necroptosis (Hu et al., 2025). In order to study the specific mechanism by which DM-Exos protect skin cells from death, we pretreated cells with DM-Exos, specific inhibitors of apoptosis (Z-VAD-FMK), necroptosis (Nec-1), and ferroptosis (Fer-1) for 12 h before UVB exposure and maintained them in inhibitor-containing medium for an additional 48 h post-irradiation. CCK-8 assays confirmed that UVB-exposed cells had significantly reduced viability, but co-treatment with DM-Exos and the ferroptosis inhibitor Fer-1 provided the most effective rescue (Figure 1G). These findings suggest that donkey milk-derived exosomes protect skin cells from UVB-induced damage, likely through ferroptosis inhibition.

### 3.2 Donkey milk-derived exosomes suppress UVB-induced ferroptosis in skin cells

**FIGURE 2:**
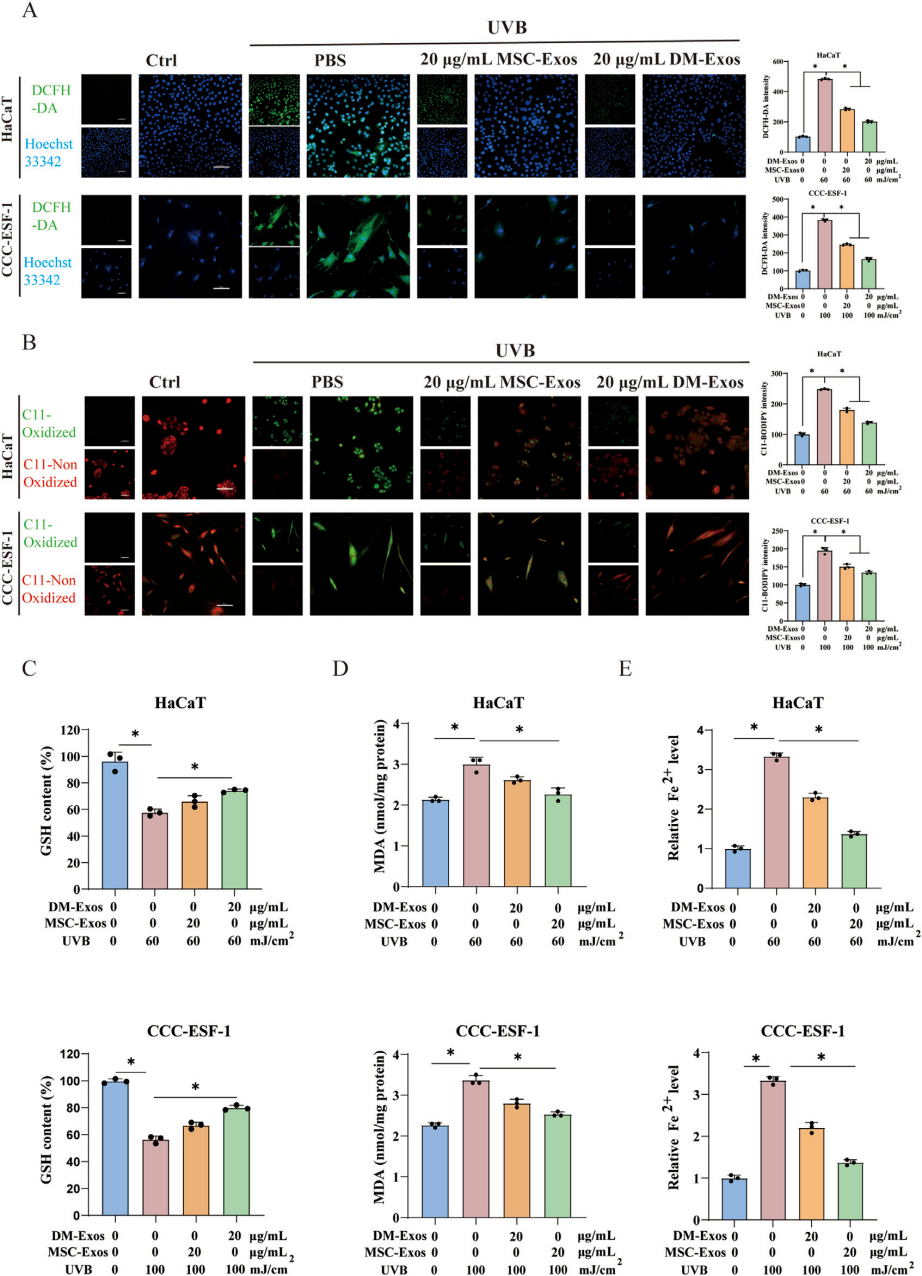
Donkey milk-derived exosomes reduce UVB-induced oxidative stress and lipid peroxidation in skin cells. **(A)** Intracellular ROS levels in UVB-irradiated HaCaT and CCC-ESF-1 cells treated with MSC-Exos or DM-Exos. **(B)** Lipid peroxidation levels in UVB-irradiated HaCaT and CCC-ESF-1 cells treated with MSC-Exos or DM-Exos. **(C)** The levels of GSH in HaCaT and CCC-ESF-1 cells treated with MSC-Exos or DM-Exos after UVB irradiation. **(D)** MDA levels in HaCaT and CCC-ESF-1 cells treated with MSC-Exos or DM-Exos after UVB irradiation. **(E)** Intracellular Fe^2+^ contents were in HaCaT and CCC-ESF-1 cells assessed using Cell Ferrous Iron Fluorometric Assay Kit. *Data are presented as mean ± SD, with statistical significance indicated by P < 0.05, as determined by one-way ANOVA.

To further investigate whether DM-Exos’ protective effects were mediated through modulation of ferroptosis—a unique form of regulated cell death driven by lipid peroxidation, we quantified intracellular ROS and LipoROS levels in UVB-exposed skin cells. Compared to control, UVB irradiation significantly increased ROS and LipoROS levels, demonstrating its dual induction of oxidative stress and ferroptosis signaling. However, DM-Exos and MSC-Exos treatment effectively reversed this increase, with DM-Exos providing stronger protection (Figures 2A,B), indicating DM-Exos mitigated UVB-induced oxidative damage in skin cells, likely by inhibiting ferroptosis.

Building upon the observed elevation in ROS and lipid peroxides, we next examined the status of cellular antioxidant defenses. As the primary endogenous antioxidant, GSH plays a key role in counteracting lipid peroxidation through GPX4-mediated conversion to less reactive lipid alcohols (LOH) (Jiang et al., 2021). UVB irradiation significantly reduced intracellular GSH levels, indicating increased oxidative stress. Intriguingly, DM-Exos and MSC-Exos treatment restored GSH levels, with DM-Exos showing stronger protective effects (Figure 2C). Additionally, we measured MDA, a lipid peroxidation end product disrupting cell membranes (Wang et al., 2023), using an MDA colorimetric assay kit. UVB exposure led to a marked increase in MDA levels, but DM-Exos treatment significantly reversed this effect (Figure 2D). Moreover, we investigated the intracellular iron accumulation in HaCaT and CCC-ESF-1 cells. We found that Fe^2+^ levels were markedly increased upon exposure to UVB, which was notably rescued by exosome treatment (Figure 2E). These findings indicate that DM-Exos mitigate UVB-induced oxidative damage by maintaining GSH levels and reducing lipid peroxidation.

### 3.3 Donkey milk-derived exosomes preserves mitochondrial membrane potential and morphology in UVB-irradiated skin cells

**FIGURE 3:**
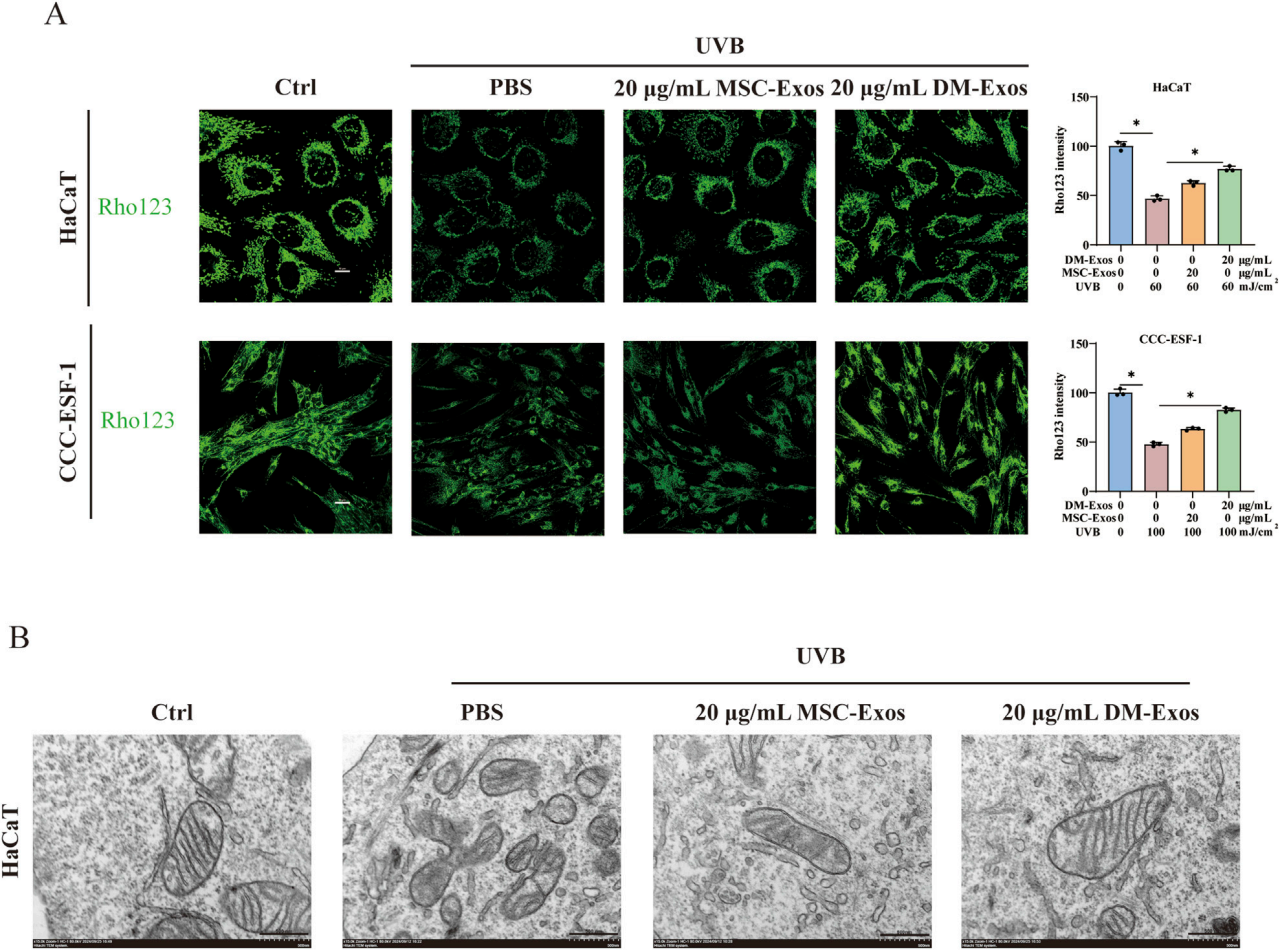
Donkey milk-derived exosomes preserves mitochondrial membrane potential and morphology in UVB-irradiated skin cells. **(A)** Mitochondrial membrane potential in UVB-irradiated HaCaT and CCC-ESF-1 cells treated with MSC-Exos or DM-Exos. **(B)** Mitochondrial ultrastructure in UVB-irradiated HaCaT cells treated with DM-Exos and MSC-Exos. *Data are presented as mean ± SD, with statistical significance indicated by P < 0.05, as determined by one-way ANOVA.

Ferroptosis is characterized by mitochondrial damage, including membrane potential loss, cristae disruption, and outer membrane rupture (Chen et al., 2021). To assess mitochondrial function, we used the Rhodamine 123 (Rho123) probe to measure mitochondrial membrane potential. UVB irradiation caused a significant reduction in membrane potential, accompanied by persistent opening of the mitochondrial permeability transition pore, leading to weakened fluorescence intensity. DM-Exos treatment restored mitochondrial membrane potential, indicating a protective effect (Figure 3A). To further evaluate mitochondrial integrity, we performed transmission electron microscopy (TEM). UVB exposure induced mitochondrial shrinkage, cristae loss, and outer membrane rupture in HaCaT cells, whereas DM-Exos and MSC-Exos treatments partially preserved mitochondrial structure (Figure 3B). These findings suggest that Donkey Milk-derived Exosomes protect mitochondria from UVB-induced damage, likely through ferroptosis inhibition.

### 3.4 Donkey milk-derived exosomes protect against changes in the expression levels of ferroptosis-related genes in skin cells induced by UVB irradiation

**FIGURE 4:**
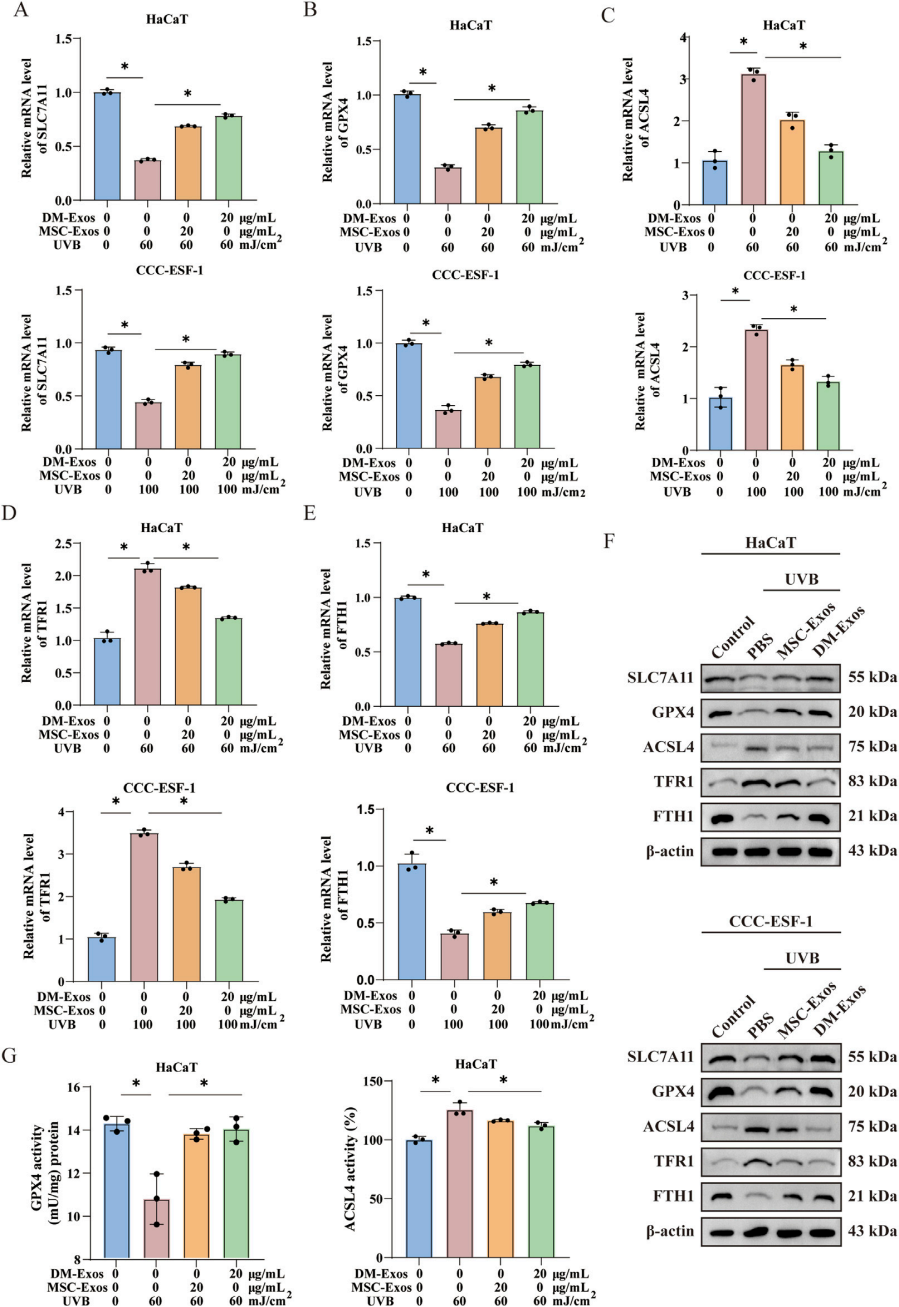
Donkey milk-derived exosomes protect against changes in the expression levels of ferroptosis-related genes in skin cells induced by UVB irradiation. **(A)** The mRNA expression level of SLC7A11 in HaCaT and CCC-ESF-1 cells treated with DM-Exos after UVB irradiation. **(B)** The mRNA expression level of GPX4 in HaCaT and CCC-ESF-1 cells treated with DM-Exos after UVB irradiation. **(C)** The mRNA expression level of ACSL4 in HaCaT and CCC-ESF-1 cells treated with DM-Exos after UVB irradiation. **(D)** The mRNA expression level of TFR1 in HaCaT and CCC-ESF-1 cells treated with DM-Exos after UVB irradiation. **(E)** The mRNA expression level of FTH1 in HaCaT and CCC-ESF-1 cells treated with DM-Exos after UVB irradiation. **(F)** The expression level of ferroptosis-related proteins in HaCaT and CCC-ESF-1 cells treated with different concentrations of DM-Exos and MSC-Exos after UVB irradiation. **(G)** GPX4 and ACSL4 activity measured in HaCaT cells treated with different concentrations of DM-Exos and MSC-Exos after UVB irradiation. *Data are presented as mean ± SD, with statistical significance indicated by P < 0.05, as determined by one-way ANOVA.

**FIGURE 5:**
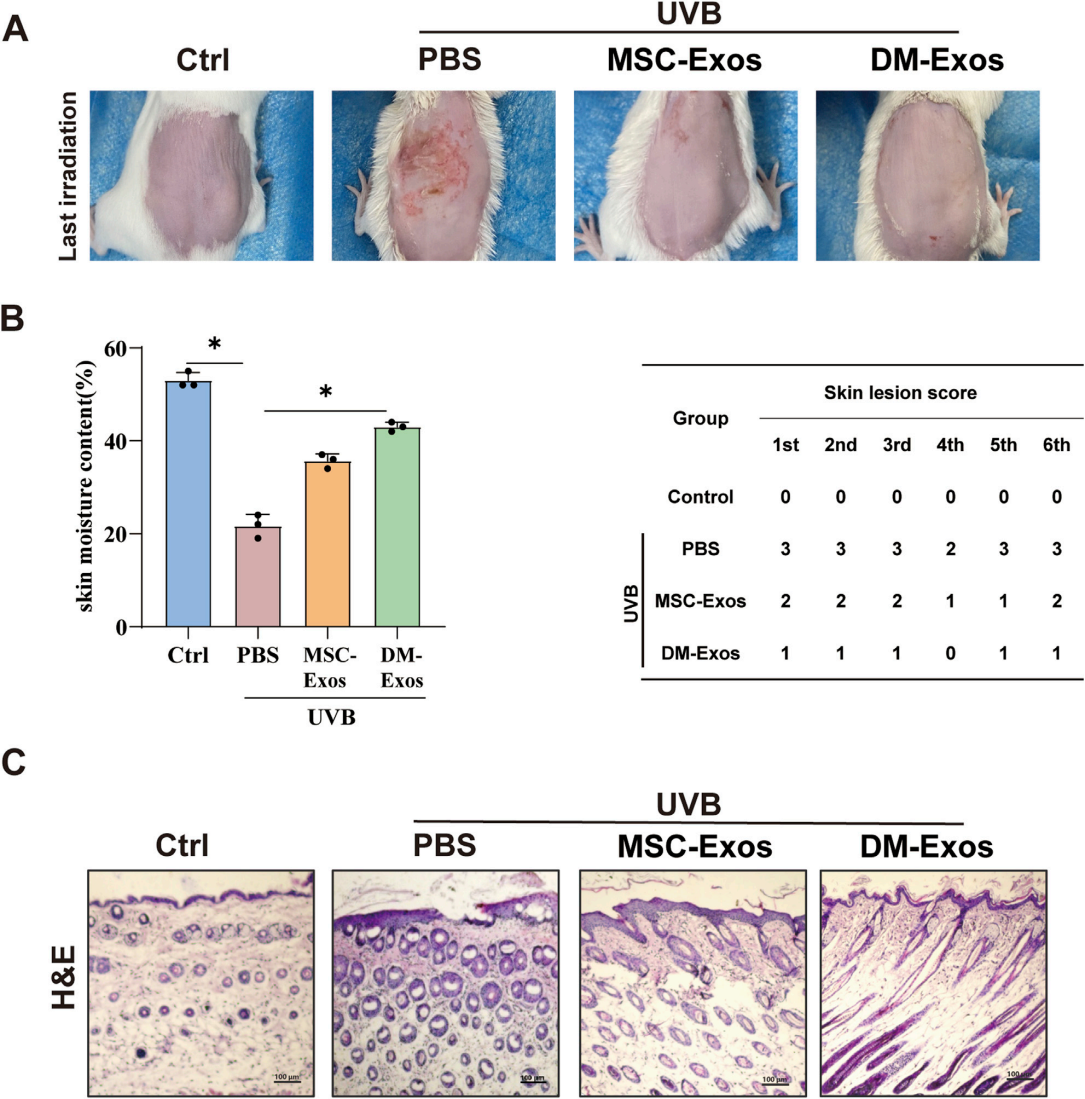
Donkey milk-derived exosomes reduce UVB-induced skin damage and improve skin hydration in mice. **(A)** Representative images of dorsal skin from different treatment groups. Mice were exposed to UVB irradiation and treated with MSC-Exos or DM-Exos (200 ug/kg). **(B)** Quantification of skin hydration and lesion severity. Skin moisture content (%) was measured across all groups. **(C)** Histological analysis of skin thickness.

Having established that donkey milk-derived exosomes mitigate oxidative stress and lipid peroxidation, we proceeded to examine their regulatory effects on core ferroptosis-related genes. Ferroptosis is governed by a network of critical regulators, including GPX4 (Deng et al., 2023), SLC7A11 (Rochette et al., 2022), FTH1 (Tia et al., 2020), TFR1 (Wang et al., 2024) and ACSL4 (Ding et al., 2023), and the results showed that UVB irradiation significantly reduced the mRNA levels of GPX4, SLC7A11, and FTH1, while increasing TFR1 and ACSL4 expression (Figures 4A–E). Treatment with DM-Exos more effectively reversed these changes, compared with MSC-Exos (Figures 4A–E). Additionally, the protein expression levels detected by Western blot analysis were consistent with the gene expression data (Figure 5F). The direct measurement of GPX4 and ACSL4 activity serves as a more definitive functional indicator of the ferroptotic status. Our data demonstrated a significant decrease of GPX4 activity concomitantly with an increase in ACSL4 activity upon UVB induction. Importantly, exosome treatment effectively restored GPX4 activity and suppressed ACSL4 activity (Figure 4G). These results suggest that donkey milk-derived exosomes help prevent UVB-induced changes in ferroptosis-related gene expression in skin cells.

### 3.5 Donkey milk-derived exosomes mitigates UVB-induced skin damage in mice

To evaluate the *in vivo* protective effects of donkey milk-derived exosomes against UVB-induced skin damage, we established a photoaging model in 4–6 week-old mice subjected to progressively increasing UVB irradiation (initial dose: 300 mJ/cm^2^, escalating weekly to 1,200 mJ/cm^2^ over 4 weeks and maintained until week 8). Compared to the control group, UVB-exposed mice developed visible skin damage, including roughness, dryness, reduced elasticity, and crusting. DM-Exos treatment at doses of 200 μg/kg exhibited more potently therapeutic efficacy, compared with MSC-Exos (Figure 5A). Notably, UVB-exposed mice displayed pronounced erythema and scabbing, whereas DM-Exos treatment markedly ameliorated these effects, with higher DM-Exos concentrations providing greater protection (Figure 5A). To quantify skin hydration and damage, we measured moisture levels and skin damage scores across all groups. UVB exposure significantly reduced moisture content and increased damage scores, while DM-Exos treatment restored moisture levels and alleviated skin damage in a dose-dependent manner (Figure 5B). Since UVB-induced inflammation and edema often result in increased skin thickness, we performed H&E staining to evaluate histological changes. UVB exposure caused a significant increase in skin thickness, but DM-Exos treatment reduced epidermal thickening, indicating a protective effect (Figure 5C). These findings demonstrate that DM-Exos protect against UVB-induced skin damage in mice by preserving skin hydration, reducing skin proliferation, and restoring skin barrier function, highlighting its potential as a therapeutic agent for UVB-induced photoaging.

### 3.6 Functional validation of donkey milk-derived exosomes for attenuating UVB-induced ferroptosis

**FIGURE 6:**
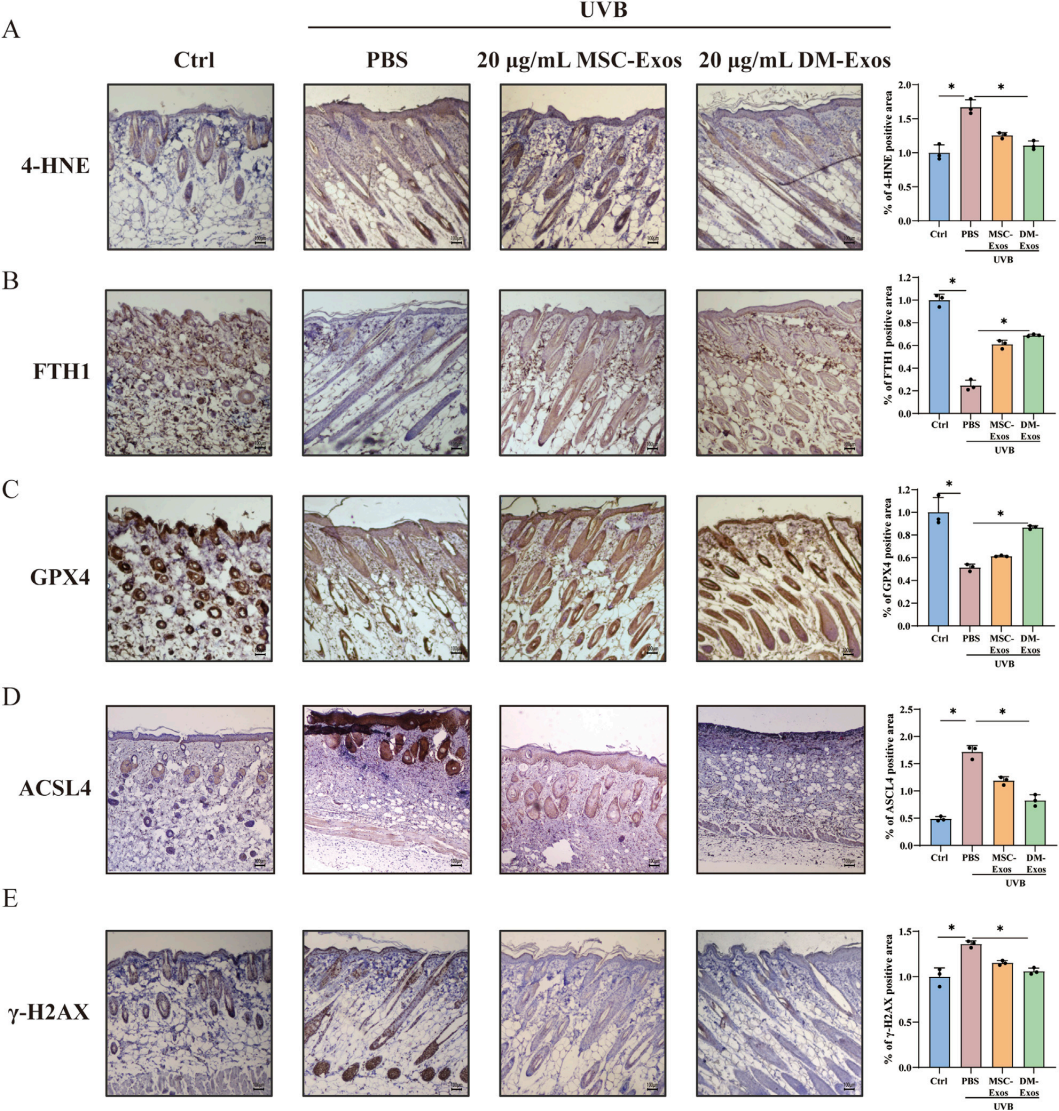
Donkey milk-derived exosomes inhibit UVB-induced ferroptosis and DNA damage in mouse skin. **(A–E)** Representative IHC images and quantification of 4-HNE, FTH1, GPX4, ACSL4 and γ-H2AX in the control, UVB-radiation, MSC-Exos or DM-Exos groups. Data are presented as mean ± SD. *P < 0.05 was considered statistically significant.

To functionally validate the protective effects of donkey milk-derived exosomes against UVB-induced ferroptosis, we systematically evaluated ferroptosis-related biomarkers (4-HNE, GPX4, FTH1, and ACSL4) and DNA damage markers (γ-H2AX) in mouse skin, and mitochondrial integrity in skin cells. IHC staining showed that 4-HNE, which is related with DNA damage and lipid membrane disruption (Ji et al., 2022), was significantly elevated in UVB-irradiated skin compared to controls. DM-Exos treatment markedly reduced 4-HNE levels, compared with MSC-Exos, suggesting that DM-Exos inhibited UVB-induced lipid peroxidation (Figure 6A). We further assessed iron homeostasis by measuring FTH1, and the results showed that UVB irradiation led to a significant reduction in FTH1 expression. DM-Exos treatment more effectively restored FTH1 expression, suggesting that DM-Exos help maintain iron balance and mitigates ferroptosis progression (Figure 6B). Next, we found that UVB exposure significantly downregulated GPX4 and upregulated ACSL4 expression in mouse skin (Figures 6C,D), whereas DM-Exos or MSC-Exos treatment reversed these changes, with DM-Exos dose producing stronger effects, further supporting the role of donkey milk-derived exosomes in ferroptosis inhibition.

To investigate DNA damage repair, we analyzed γ-H2AX expression, a marker of DNA double-strand breaks (Ibuki et al., 2015). UVB irradiation significantly increased γ-H2AX expression, whereas DM-Exos treatment reduced γ-H2AX levels, indicating that DM-Exos help mitigate UVB-induced DNA damage (Figure 6E). Collectively, these results demonstrate that DM-Exos protect against UVB-induced ferroptosis in mouse skin by reducing lipid peroxidation, restoring iron homeostasis, and enhancing GPX4 expression.

## 4 Discussion

**FIGURE 7:**
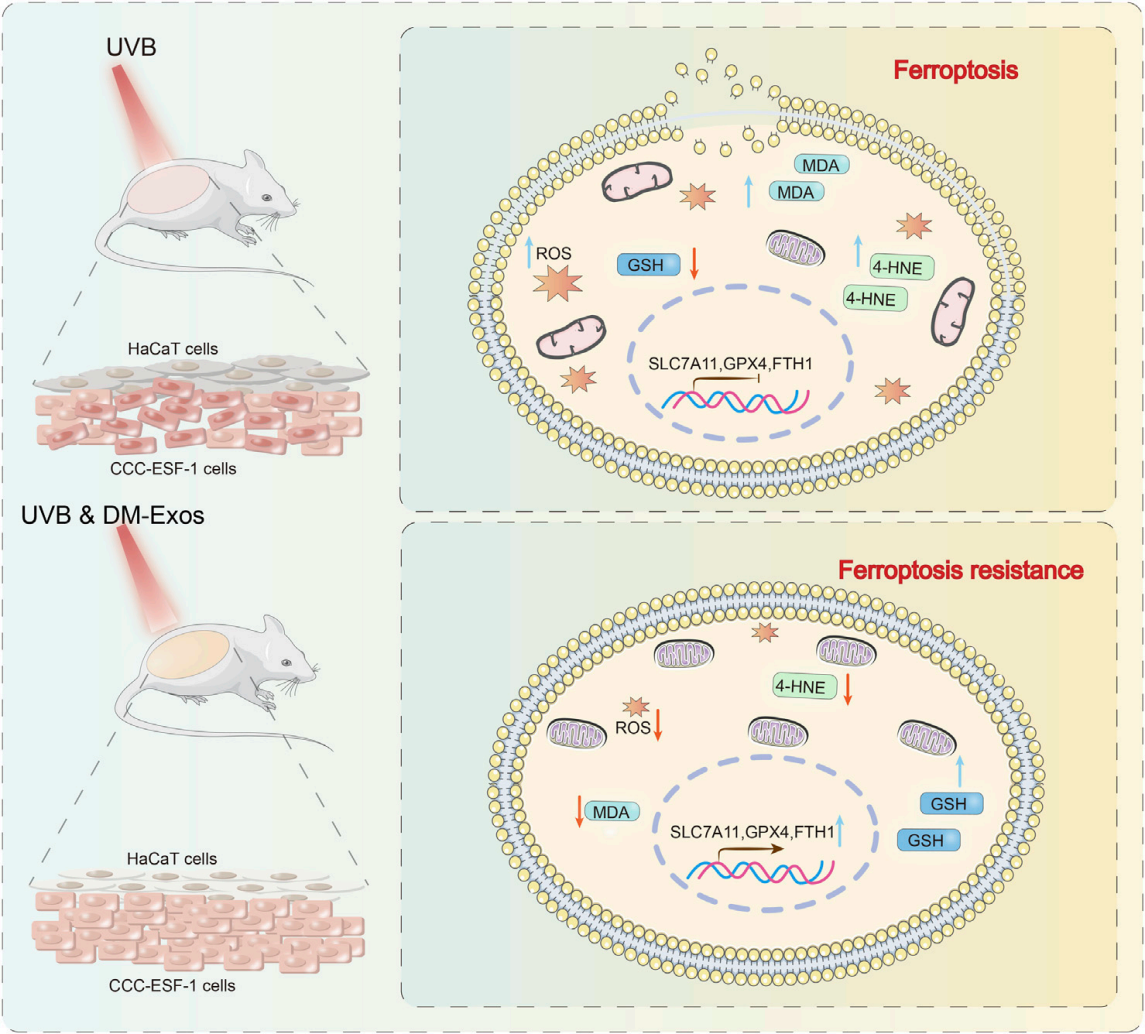
Schematic diagram of donkey milk-derived exosomes protecting against UVB-induced cellular ferroptosis. In skin cells, UVB exposure increases levels of ROS, LipoROS, and MDA, while reducing GSH levels. DM-Exos treatment significantly reverses these changes by reducing ROS, LipoROS, and MDA levels and restoring GSH content in UVB-irradiated HaCaT and CCC-ESF-1 cells. In the UVB-irradiated Balb/c mouse model, the expression levels of 4-HNE, GPX4, and FTH1 were altered, indicative of ferroptosis activation. DM-Exos treatment reversed these changes.

Exosomes, small vesicles released by cells via exocytosis, contain proteins, lipids, RNA, and other biomolecules and facilitate intercellular communication (Gurunathan et al., 2019). Recent studies have elucidated the antioxidant properties of exosomes, demonstrating their ability to regulate intracellular redox balance and enhance the cellular antioxidant defense system by reducing ROS production, thereby exerting anti-inflammatory and cytoprotective effects. UVB radiation induces inflammatory and oxidative stress responses, contributing to skin damage, yet the underlying therapeutic agents are not fully understood. In this study, we demonstrated that DM-Exos exert protective effects against UVB-induced ferroptosis in HaCaT and CCC-ESF-1 cells. Our findings revealed that subcutaneous application of DM-Exos effectively reduced epidermal thickening, oxidative stress, and ferroptosis in UVB-irradiated mouse skin, suggesting its potential as a therapeutic intervention for photodamage (Figure 7).

Ferroptosis is a kind of cell death that differs from apoptosis in that it is driven by iron-dependent lipid reactive oxygen species (Zhou et al., 2024). It is not difficult to see that the primary cause of ferroptosis is an overabundance of lipid ROS, which is brought on by an imbalance between the lipid oxidation and antioxidant system (Jiang et al., 2021). Excessive lipid ROS cause damage to a number of crucial functional compounds within cells, a process that is similar to that of UVB-induced oxidative stress injury and skin photoaging (Cui et al., 2022). Therefore, UVB-induced skin photoaging may also entail ferroptosis. As expected, our study showed that UVB exposure significantly increased intracellular ROS, LipoROS, and MDA levels while reducing GSH content and mitochondrial membrane potential, confirming UVB-induced ferroptosis activation (Figures 2, 3). Notably, DM-Exos exhibited comparable or superior efficacy to mesenchymal stem cell-derived exosomes (MSC-Exos), an established positive control in photoprotection, in restoring redox homeostasis and suppressing ferroptotic markers, underscoring the potent bioactivity of DM-Exos (Figures 2–4). Importantly, this protective activity was not cell-type-specific. Although HaCaT and CCC-ESF-1 cells exhibited differential sensitivity to UVB, DM-Exos consistently ameliorated ferroptosis hallmarks in both models, reinforcing its potential as a universal agent against UVB-induced redox imbalance in skin.

Given that ferroptosis plays a pivotal role in UVB-induced skin damage, inflammation, and aging (Xu et al., 2025), our findings suggest that DM-Exos could serve as a natural photoprotective agent. The ability of DM-Exos to mitigate oxidative stress and ferroptosis further supports its potential applications in both clinical dermatology and cosmetic formulations. Although our study provides compelling evidence of DM-Exos’ protective effects against UVB-induced ferroptosis, several questions remain. Future studies should investigate the specific molecular pathways by which DM-Exos regulate ferroptosis-related genes and antioxidant responses. While we observed protective effects *in vitro* and in short-term animal models, long-term studies are necessary to assess whether DM-Exos can provide sustained protection against UV-induced aging and skin disorders. Additionally, further research is needed to explore the optimal formulation, dosage, and delivery methods of DM-Exos for applications in human skin protection.

Our findings provide new insights into DM-Exos’ role in protecting against UVB-induced ferroptosis. The ability of DM-Exos to counteract lipid peroxidation, restore antioxidant balance, and maintain mitochondrial function underscores its potential as a therapeutic agent for preventing UV-induced photoaging and skin inflammation. These results pave the way for future studies exploring DM-Exos’ applications in dermatology and skincare formulations.

## Data Availability

The raw data supporting the conclusions of this article will be made available by the authors, without undue reservation.
