# Supplementary material for: Donkey milk-derived exosomes protect against UVB irradiation-induced ferroptosis in skin cells: in vitro and in vivo evidence
Source: Front Pharmacol. 2026 Jan 2;16:1683253. doi: 10.3389/fphar.2025.1683253 (PMC12808391; doi:10.3389/fphar.2025.1683253)
Supplement: Supplementary file 1 [file Table1.docx]

**Table S1 Primer sequences**

| Gene |  |  | Primer sequences |
| --- | --- | --- | --- |
| β-actin |  | Forward primer: | 5’-AGCCTTCCTTCCTGGGCAT-3’ |
|  |  | Reverse primer: | 5’-TGATCTTCATTGTGCTGGGTG-3’ |
| SLC7A11 |  | Forward primer: | 5’-GGTCCATTACCAGCTTTTGTACG-3’ |
|  |  | Reverse primer: | 5’-AATGTAGCGTCCAAATGCCAG-3’ |
| GPX4 |  | Forward primer: | 5’-GAGGCAAGACCGAAGTAAACTAC-3’ |
|  |  | Reverse primer: | 5’-CCGAACTGGTTACACGGGAA-3’ |
| FTH1 |  | Forward primer: | 5’-ACGTTTACCTGTCCATGTCTTACT-3’ |
|  |  | Reverse primer: | 5’-AAGGAAGATTCGGCCACCTC-3’ |
| TFR1 |  | Forward primer: | 5’-CTGGCTCGGCAAGTAGATGG-3’ |
|  |  | Reverse primer: | 5’-CCAAGTAGCCAATCATAAATCCAAT-3’ |
| ACSL4 |  | Forward primer: | 5’-GCTATCTCCTCAGACACACCGA-3’ |
|  |  | Reverse primer: | 5’-AGGTGCTCCAACTCTGCCAGTA-3’ |
