# Supplementary material for: Donkey milk-derived exosomes protect against UVB irradiation-induced ferroptosis in skin cells: in vitro and in vivo evidence
Source: Front Pharmacol. 2026 Jan 2;16:1683253. doi: 10.3389/fphar.2025.1683253 (PMC12808391; doi:10.3389/fphar.2025.1683253)
Supplement: Supplementary file 2 [file Table2.docx]

**Table S2. List of antibodies used in this study**

| Antibodye | Mmanufacturere | Catalog | Application. |
| --- | --- | --- | --- |
| ACSL4 | ABclonal | A22901 | 1:10000 for WB  1:200 for IHC |
| SLC7A11 | Proteintech | 26864-1-AP | 1:2000 for WB |
| GPX4 | ABclonal | A1933 | 1:10000 for WB  1:200 for IHC |
| TFR1 | Abcam | ab214039 | 1:1000 for WB |
| FTH1 | ABclonal | A19544 | 1:2000 for WB  1:200 for IHC |
| β-actin | ABclonal | AC026 | 1:10000 for WB |
| 4-HNE | Abcam | ab46545 | 1:200 for IHC |
| γ-H2AX | Abcam | ab81299 | 1:200 for IHC |
